# Red Cabbage Modulates Composition and Co-Occurrence Networks of Gut Microbiota in a Rodent Diet-Induced Obesity Model

**DOI:** 10.3390/foods13010085

**Published:** 2023-12-26

**Authors:** Yanbei Wu, Mengmeng Xin, Quynhchi Pham, Yu Gao, Haiqiu Huang, Xiaojing Jiang, Robert W. Li, Liangli Yu, Yaguang Luo, Jing Wang, Thomas T. Y. Wang

**Affiliations:** 1 China-Canada Joint Lab of Food Nutrition and Health (Beijing), Beijing Technology & Business University, Beijing 100084, China; yanbeiwu@btbu.edu.cn (Y.W.); xmm298621@163.com (M.X.); gy1678224115@163.com (Y.G.); wangjing@th.btbu.edu.cn (J.W.); 2 Diet Genomics and Immunology Laboratory, BHNRC, ARS, USDA, Beltsville, MD 20705, USA; quynhchi.pham@usda.gov; 3 Department of Nutrition and Food Science, University of Maryland, College Park, MD 20742, USAcharlenejiang1001@gmail.com (X.J.); lyu5@umd.edu (L.Y.); 4 Animal Parasitic Diseases Laboratory, BARC, ARS, USDA, Beltsville, MD 20705, USA; robert.li@usda.gov; 5 Food Quality Laboratory, BARC, ARS, USDA, Beltsville, MD 20705, USA; yaguang.luo@usda.gov

**Keywords:** red cabbage, mice obesity model, gut microbiota, 16S rRNA sequencing

## Abstract

Red cabbage (RC), a cruciferous vegetable rich in various bioactive substances, can significantly reduce the risk factors of several non-communicable diseases, but the mechanism underlying the biological effects of RC remains unclear. Furthermore, mechanisms that operate through the regulation of gut microbiota also are not known. Given the relationships between diet, gut microbiota, and health, a diet-induced mice obesity model was used to elucidate the influence of RC on gut microbial composition and bacteria–bacteria interactions in mice. After 24 h of dietary intervention, a high-fat (HF) diet with the intake of RC led to increased Firmicutes/Bacteroidetes (F/B) ratios in the feces of mice. RC also reduced the relative abundance of *Bifidobacteria*, *Lactobacillus*, and *Akkermansia muciniphila* in mice fed a low-fat (LF) diet. After 8-weeks of dietary intervention, RC significantly changed the structure and the ecological network of the gut microbial community. Particularly, RC inhibited an HF-diet-induced increase in *AF12* in mice, and this genus was positively correlated with body weight, low-density lipoprotein level, and fecal bile acid of mice. Unclassified *Clostridiales*, specifically increased via RC consumption, were also found to negatively correlate with hepatic free cholesterol levels in mice. Overall, our results demonstrated that RC modulating gut microbial composition and interactions are associated with the attenuation of HF-diet-induced body weight gain and altered cholesterol metabolism in mice.

## 1. Introduction

The human digestive tract commonly hosts a diverse range of microorganisms, including bacteria, fungi, viruses, and parasites, collectively known as the gut microbiota. This microbiota, comprising over 100 billion to 100 trillion microorganisms, plays a crucial role in maintaining the dynamic equilibrium of human health and diseases [1,2,3]. The microorganisms in the gut have a significant impact on the metabolism, immune system, and overall physiology of the human host. Numerous research investigations have indicated that imbalances in the gastrointestinal microbiome can play a crucial role in the emergence of various ailments, including metabolic syndrome (excessive weight and associated conditions), high blood pressure, and depressive disorders [4,5,6]. Obesity is currently one of the most widespread public health issues globally among chronic diseases. Projections suggest that over 1 billion individuals will face the risk of obesity by 2030 [7]. Additionally, visceral obesity contributes significantly to chronic conditions like type 2 diabetes, hepatic steatosis, and cardiovascular disease [8,9]. Studies in epidemiology have indicated that dietary interventions play a crucial role in combating obesity and its associated illnesses, and can also impact the makeup of the gut microbiome [10,11]. According to reports, people with varying eating habits possess distinct microbiomes. Consuming diets rich in sugar and fat can disturb bacterial metabolism and balance, resulting in microbiota dysbiosis, obesity, and metabolic syndrome [12]. On the other hand, the intake of phytonutrients that are abundant in vitamins, polyphenols, flavonoids, and other compounds can enhance the distribution of lipids and regulate the imbalance of gut ecology in the body. As a result, this can contribute to the prevention and treatment of different metabolic disorders [13,14]. Furthermore, alterations in dietary habits can swiftly lead to modifications in the makeup of the intestinal microbiota [15].

Red cabbage (RC), a vegetable of the Brassica genus, is a readily available consumer dietary product that is rich in purported health-promoting bioactives [16]. RC has a higher level of polyphenols and antioxidant activity compared to white cabbage [17]. The majority of polyphenols do not get absorbed in the small intestine; instead, they go into the colon where they alter the makeup of the gut microbiota. This alteration promotes the growth of helpful bacteria while suppressing harmful bacteria, ultimately enhancing the availability of polyphenols [18]. Other well-known bioactive compounds in RC, such as glucosinolates (GSLs) that are stable in the natural matrix but readily hydrolyzed by endogenous mustard enzymes (EC 3.2.1.147), activate mechanical actions when consumed, to produce compounds such as isothiocyanates (ITC) and indole [19]. According to previous reports, indole derived from GSL plays a role in preserving the natural defense of the human gut, activating aryl hydrocarbon and pregnane X receptors to exert anti-inflammatory effects, consequently impacting the functioning of the immune system [20,21]. The positive impacts of these effects on intestinal health are substantial, and they hold immense promise for treating inflammatory bowel disease and neurodegenerative disorders [22,23]. We and others have also reported that, in rodent models, feeding RC or an RC extract containing the bio-actives results in lowering blood lipids, cholesterol, cardiovascular disease risk factors, and promoting liver health [24]. We also reported that indole-3-carbinol, a dietary digestive derivative of GSL, can modulate the gut microbiome and is associated with attenuation of tumor xenograft growth [25]. Nevertheless, even though the health benefits of RC are well-known, there is still uncertainty regarding its influence on the gut microbiome as a dietary component and its potential impact on overall well-being.

We hypothesized that consumption of RC can modulate the gut microbiome and provide a protective effect on health. The present investigation aims to expand our prior research on the impact of RC on health to clarify: (1) if RC intake can influence the gut microbiome and, if it does, what modifications occur and (2) if alterations in the gut microbiome are linked to the reduction in disease risk factors. We utilized a mice model of obesity induced via high fat intake and implemented a factorial design of 2 × 2 (level of fat and with or without RC). We used 16S rRNA sequencing analysis and ecological network analysis to identify specific genera that cause alterations in gut microbiota structure and microbial symbiosis patterns. An AI (Random Forest)-assisted algorithm was used to elucidate the relationship between microbiome changes and obesity-related risk factors to address the questions raised. The findings of our research offer fresh mechanistic proof that RC has the potential to decrease the likelihood of metabolic disorders, like obesity, through the regulation of the gut microbiota.

## 2. Materials and Methods

### 2.1. Establishment of Mice Obesity Model

A total of forty male C57BL/6NCr mice, aged around 5 weeks and weighing approximately 20 g, were acquired from Charles River Laboratories (National Cancer Institute, Frederick, MD, USA). These mice were divided randomly and equally into four groups (*n* = 10 in each group), individually housed in cages with filtered lids, and subjected to a 12 h light/dark cycle. Mice were provided with standard rodent nourishment and sufficient water for one week before commencing the experiment. Each group was assigned to a different diet: (1) LF diet (LF, 10 kcal% fat diets); (2) LF diet with the addition of RC powder (LFRC, 10 kcal% fat diets containing 10.9 g/kg diet); (3) HF diet (HF, 45 kcal% fat diets); and (4) HF diet with the addition of RC powder (HFRC, 45 kcal% fat diets containing 10.9 g/kg diet). Data such as the mice’s body weight and food intake were recorded weekly throughout the 8-week experimental cycle. The local market provided recently purchased RC, which was subsequently frozen in liquid nitrogen for 48–72 h. After being frozen, the RC samples were grounded and incorporated into the diet in the form of a dry powder. The experimental diets were formulated and pelleted by Research Diets (New Brunswick, NJ, USA). The dietary supplement for the mice was adjusted based on an equivalent of 200 g of vegetables per day for a 60 kg human, using the appropriate dose conversion formula suggested by Reagan-Shaw et al. [26]. The animal experiment was conducted following established ethical guidelines and approved by the Beltsville Area Animal Care and Use Committee of the United States Department of Agriculture (protocol # 14-006).

### 2.2. Measurement of Physiological Indicators in Mice Samples

Mouse plasma lipoprotein levels were assessed through size-exclusion chromatography (Agilent Technologies, Santa Clara, CA, USA); amounts of triacylglycerol in the liver were tested using a commercial kit (Triglyceride-SL, Sekisui Diagnostics PEI Inc., Charlottetown, Canada); free cholesterol and total cholesterol in liver were determined using the Amplex red cholesterol assay kit (Invitrogen, Carlsbad, CA, USA), and concentrations of fecal bile acid were quantified by using a commercial kit (Cell Biolabs Inc., San Diego, CA, USA). The data for these physiological indicators were obtained from our previous study [24].

### 2.3. Fecal DNA Extraction and Quantitative PCR Analysis

Mice fecal samples were collected 24 h after different dietary treatments, homogenized at 7500 rpm for 1 min using a Precellys (Bertin Technologies, Villeurbanne, France), and bacterial DNA was extracted from mice feces using the QIAamp DNA Stool Mini kit. The DNA concentration in the end solution was determined by measuring its absorbance at 260 nm and subsequently diluted step by step to reach a concentration of 10 ng/μL. Quantitative real-time PCR analyses were conducted using an Applied Biosystems™ 7900T Real-Time PCR System (Forest City, CA, USA). The PCR reaction consisted of 10 μL of SYBR^®^ Green Real-Time PCR Master Mix, 0.25 μL of 500 nM custom primers (refer to Table S1 in the electronic Supplementary Information), 4.5 μL of water, and 5 μL of DNA.

### 2.4. Cecum DNA Extraction and 16S rRNA Sequencing

Following 8 weeks, the contents of the mice’s cecum were gathered, promptly frozen in liquid nitrogen, and subsequently stored at −80 °C for preservation. The QIAamp Fast DNA Stool Mini Kit was utilized to extract total DNA from the cecum samples, followed by assessing the purity and concentration of the obtained DNA using a Nanodrop 8000 spectrophotometer (Thermo Scientific, Wilmington, DE, USA). PCR amplification of the 16S rRNA gene V3–V4 variable region of the samples was performed with the following PCR primers: forward primer 341/357F, CCTACGGG-NGGCWGCAG and reverse primer 805/785R, GACTACHVGGGTATCTAATCC, for 20 cycles. The amplification products were purified with the Agencourt AMPure XP beads kit and then quantified with the High Sensitivity DNA kit (Agilent, USA). The amplification products from each sample were combined in equal amounts and the library pool was sequenced using the Illumina Mi Seq Reagent Kit v3 and Illumina Mi Seq sequencer [27,28].

### 2.5. Sequence Data Analysis

The preprocessing of the sequencing data was conducted using MiSeq Control Software version 2.4.1. The integrity of the raw sequences was assessed with FastQC software; version 0.11.2. Trimmomatic (version 0.36) was employed to trim away low-quality reads and to remove the first four degenerate bases, represented as “NNNN”, from the 5′ ends of the read pairs. The cleaned paired-end reads were then merged using the join_paired_ends.py script. The sequenced sequences were bioinformatically analyzed using the QIIME1 tool, and the similarities were merged according to the “closed reference”. OTU is an artificially defined taxonomic unit used in phylogenetic studies to classify species and is the basic unit that constitutes the relative abundance of a species. The biology of each OTU was annotated using the RDP classifier tool by comparing it with the Greengene database. The samples were analyzed to determine the community composition at various taxonomic levels, including phylum, class, order, family, genus, and species. The majority of the OTUs were identifiable at the genus classification, while only a small number could be assigned to the species classification.

### 2.6. Statistical Analysis and Visualization

Evaluation of species diversity was conducted using the Microbiome Analyst platform (MicrobiomeAnalyst 2.0, http://www.microbiomeanalyst.ca, accessed on 18 January 2023), including Alpha and Beta diversity analysis, hierarchical clustering, and heat map visualization [29]. Comparisons of the relative abundance of taxonomic units between different diet groups were performed using GraphPad Prism 9 (GraphPad Software, La Jolla, CA, USA). The data were presented as means ± SD and subsequently subjected to one-way ANOVA and Tukey’s post hoc test. All findings were deemed significantly distinct at a significance level of *p* ≤ 0.05. The linear discriminant analysis (LDA) effect size (LEfSe) algorithm-based analysis of the Galaxy platform (http://huttenhower.sph.harvard.edu/galaxy/, accessed on 26 February 2023) was used to identify genus that were statistically different within groups (i.e., biomarker). Based on the distribution of the data, GraphPad Prism 9 and Pearson correlation analysis based on R (R version 3.6.1) were used to calculate the correlations between changes in genus-level relative abundance and obesity-related risk factors (such as weight gain, plasma lipoproteins, and lipid metabolites). The physiological data were collected from our previous studies [24].

A microbial co-occurrence network was constructed using the network analysis platform MENA (http://ieg4.rccc.ou.edu/mena/, accessed on 1 March 2023), as indicated by the results of the OTU analysis. The co-occurrence of species in the samples was simultaneously obtained using the random matrix theory (RMT). The main steps are as follows: firstly, the OTU tables were modified and uploaded according to the file format in MENA, the uploaded data were logarithmically normalized, the Pearson correlation was calculated and the correlation matrix was established, the system automatically generated the similarity threshold according to the random matrix principle, and the network was constructed based on this default threshold. The default threshold was used to build the network. Afterward, by selecting MENA network analysis, Cytoscape software (version 3.9.1) could be utilized to compute and display the network property parameters, thereby acquiring the network configuration and associated details. These details encompassed the count of nodes, which represented the number of species within the community (OTUs), the interconnection between nodes (whether positive or negative correlation), the clustering coefficient, and the count of species within the community (OTUs). The clustering coefficient measured the level of connectivity between a node and other nodes, while modularity described the degree of modularity in the molecular ecological network. The intra-module connectivity (Zi) and inter-module connectivity (Pi) could be used to express the roles of nodes. The Mantel test was employed to assess the significance of OTU and node connectivity in relation to the correlation between network topology and physiological traits, based on the findings of the molecular ecological network. Subsequently, the association between the colony network and the characteristic factors was determined [30].

## 3. Results

### 3.1. Effect of Consuming RC for 24 h on the Fecal Microbiota of Mice

**Figure 1:**
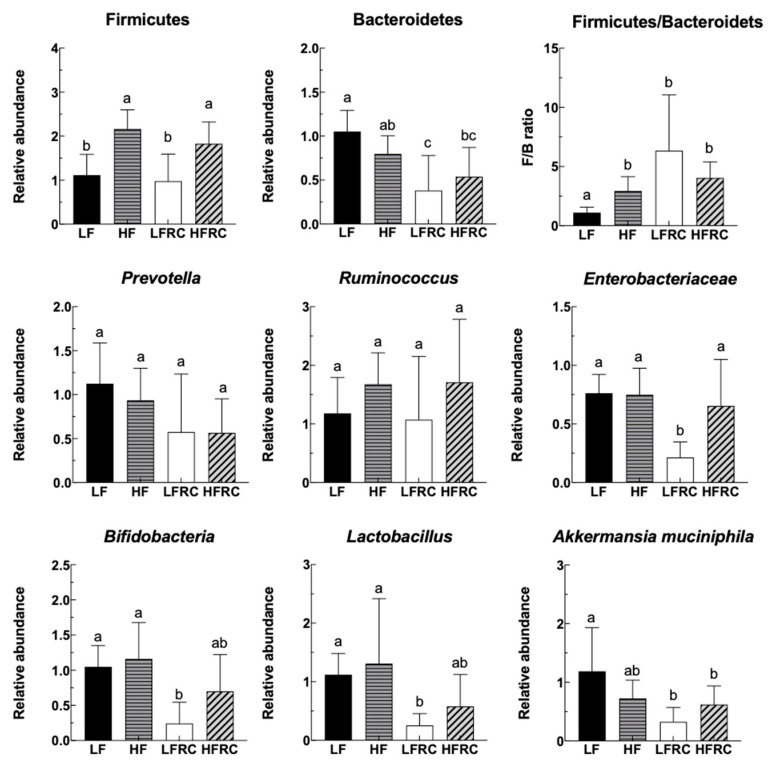
The influence of RC on the intestinal bacteria of mice. Mouse fecal samples were collected 24 h after feeding, from which bacterial DNA was extracted and quantified using qRT-PCR with specific primers. The information is displayed as mean ± SD. Statistically significant differences between groups are denoted by distinct letters, with a *p*-value less than 0.05 indicating significance.

The relative abundance of Firmicutes, Bacteroidetes, Prevotella, *Ruminococcus*, *Enterobacteriaceae*, *Bifidobacteria*, *Lactobacillus*, and *Akkermansia muciniphila* in feces was quantified using the RT-PCR method after the mice consumed various diets for 24 h. As shown in Figure 1, the LF group had a significantly lower abundance of Firmicutes in their feces compared to the HF-diet-fed mice, whereas the inclusion of RC had minimal impact on the relative abundance of Firmicutes in both the LF and HF groups. On the other hand, there was no notable variation in Bacteroidetes between the LF and HF groups. However, the presence of RC supplementation notably reduced the prevalence of Bacteroidetes in the LFRC group, whereas it did not have a significant impact on the HFRC group. Furthermore, the ratio of F/B was significantly increased in all other three groups compared to the LF group. Additionally, there were no notable distinctions observed in *Prevotella*, *Ruminococcus*, *Enterobacteriaceae*, *Bifidobacteria*, *Lactobacillus*, and *Akkermansia muciniphila* between the LF and HF groups. The impact of RC on Prevotella and *Ruminococcus* was also not significant; however, it notably decreased the prevalence in the LFRC group of *Enterobacteriaceae*, *Bifidobacteria*, and *Lactobacillus*. There was no significant difference between HF and HFRC groups for these bacteria. The LFRC group showed a significantly reduced relative abundance of *Akkermansia muciniphila* compared to the LF group when supplemented with RC.

### 3.2. Effect of Consuming RC for 8 Weeks on Microbial Diversity in the Mice Cecum

**Figure 2:**
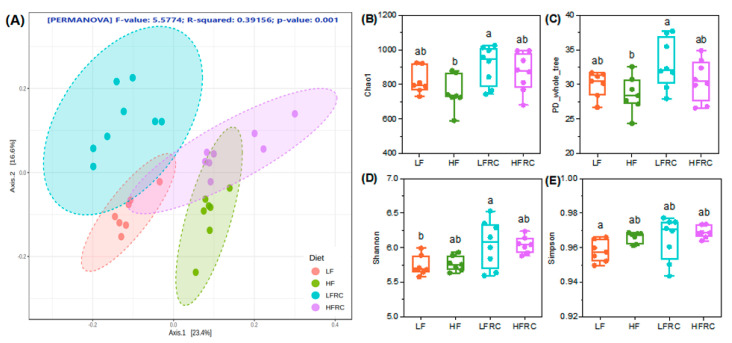
The impact of RC on the diversity of microbes in the cecum of mice that were given an LF and HF diet. Following an 8-week treatment period, the contents of the cecum in mice were gathered and the DNA of bacteria in the cecal contents was extracted for sequencing the 16s rRNA. (**A**) Nonmetric multidimensional scaling (NMDS) plot of the β diversity based on the OTU level Bray–Curtis distance matrix. The significance of clustering patterns in two-dimensional ordination diagrams was assessed using Permutation analysis of variance (PERMANOVA). (**B**–**E**) Box plot of OTU-level alpha diversity index (Chao1, PD whole tree, Shannon, and Simpson). Detailed analysis was performed by conducting statistical evaluation using one-way ANOVA followed by Tukey’s multiple comparison test. Boxes marked with different letters indicate statistically significant differences at a significance level of *p* ≤ 0.05.

The long-term effects of RC on the cecal microbiota of mice were analyzed using 16S rRNA sequencing after consuming different grouped diets for 8 weeks. Initially, the Bray–Curtis distance algorithm was employed in OTU abundance-based principal coordinate analysis (PCoA) to assess the variation in species diversity among samples. As shown in Figure 2A, the gut microbial communities of mice at different dietary levels were separated from each other, whereas when RC was added to the diet, the differences between microbial communities of LFRC and HFRC groups were reduced as indicated by overlapping circles. Furthermore, the alteration in species diversity can be evaluated by employing Chao1 and PD-whole-tree indices (Figure 2B,C); it was discovered that including RC in the diet did not have a notable impact on the alteration of species diversity in either the LF or HF groups. Also, using Shannon and Simpson indexes to indicate changes in species evenness (Figure 2D,E), it was observed that the Shannon index exhibited a significant increase in the LFRC group when compared to the LF group. However, no significant difference was observed between the HF and HFRC groups. There were no significant differences in species richness and evenness between the LF and HF diets for all indexes assessed. In general, the inclusion of RC in the diet did not have a notable impact on the gut microbiota diversity of mice following both the LF and HF diets.

### 3.3. Effect of Consuming RC for 8 Weeks on the Composition of Cecal Microbiota in Mice

**Figure 3:**
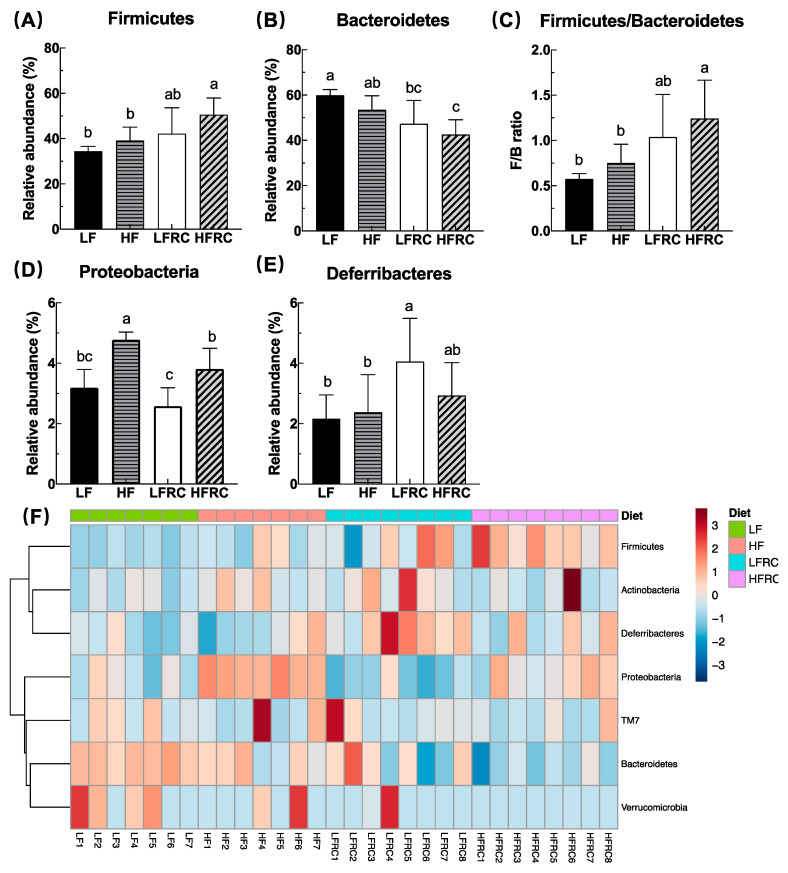
The cecal microbiota profile at the phylum level in mice under different dietary regimes shows variations. The relative percentage abundance of the predominant phyla (**A**,**B**,**D**,**E**) represents each group, where the four most abundant phyla make up at least 99% of all identified OTUs in each sample. Additionally, the ratio of Firmicutes to Bacteroidetes is provided for each group (**C**). The data are displayed as mean ± SD. Bars containing unique letters indicate statistically significant distinctions, with a *p* ≤ 0.05. Additionally, (**F**) exhibits a heatmap illustrating the relative prevalence of 16S rRNA gene sequences classified by phylum, employing hierarchical clustering analysis conducted via Microbiome Analyst utilizing the Ward clustering algorithm and the Euclidean distance metric.

To elucidate the effect of four different diets, the structure of the mouse gut microbiome was further analyzed. In all samples, a total of six phyla were detected, with the top four phyla making up over 99% of the bacterial sequences. The dominant phylum was Bacteroidetes (60%), followed by Firmicutes (34%), Proteobacteria (3%), and Deferribacteres (2%) (as shown in Table S2). As shown in Figure 3A,B, at the phylum level, the inclusion of RC in the diet led to a higher abundance of Firmicutes and a lower abundance of Bacteroidetes in the high-fat (HF) diet, which was not observed in the LF diet. In comparison, the HFRC group exhibited a notable increase in the F/B ratio when compared to both the LF and HF groups (Figure 3C). In addition, the inclusion of RC in the diet decreased the abundance of Proteobacteria in the HF diet but not in the LF diet (Figure 3D). The abundance of Deferribacteres was significantly greater in the LFRC group compared to the LF group, but there was no significant difference between the HF group and the HFRC group (Figure 3E). Figure 3F displays a heat map illustrating the distribution of species composition within the gut microbial phylum as an alternative illustration of bacterial differences. The main groups detected were Bacteroidetes, Firmicutes, Proteobacteria, Deferribacteres, TM7, Actinobacteria, and Verrucomicrobia. The specific differences mirrored Figure 3A–D.

**Figure 4:**
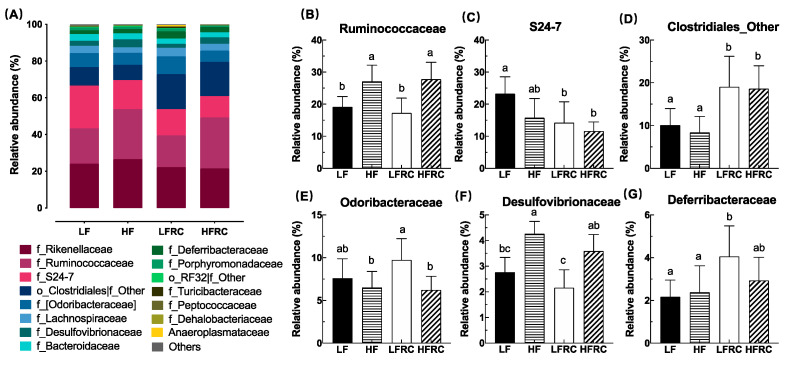
(**A**) The composition of the cecal microbiota at the family level was altered in mice on various diets. Data are presented for the top 15 families, with the collective contribution of other taxa represented as “Others”. (**B**–**G**) Display the comparative relative abundances at the family level among different microbial populations. Results are shown as mean ± SD. Bars annotated with different letters denote significant intergroup differences at *p* ≤ 0.05.

A total of 65 bacterial taxa at the level of the family were detected. To compare the variations in species composition between samples, a cumulative bar chart was utilized, and only the top 15 families account for over 99% of the bacterial sequences (Figure 4A). This chart also includes two families that are not classified, namely *Clostridiales* and *RF32* (Figure 4A). Among the first 15 families, the abundance of six families was more significantly affected by different diets (Figure 4B–F). After adding RC to the diet, the HF group exhibited significantly higher levels of *Ruminococcaceae* and *Desulfovibrionaceae* compared to the LF group, while the LFRC group showed significantly lower levels of *S24-7* compared to the LF group. In contrast, *Deferribacteraceae* was significantly higher. In the meantime, the prevalence of the LFRC and HFRC categories of Unclassifed *Clostridiales* was considerably greater in comparison to the LF and HF categories. In addition, in other HF groups, the addition of RC did not cause any significant change in abundance.

### 3.4. Identification of Microbial Biomarkers Associated with a Diet

**Figure 5:**
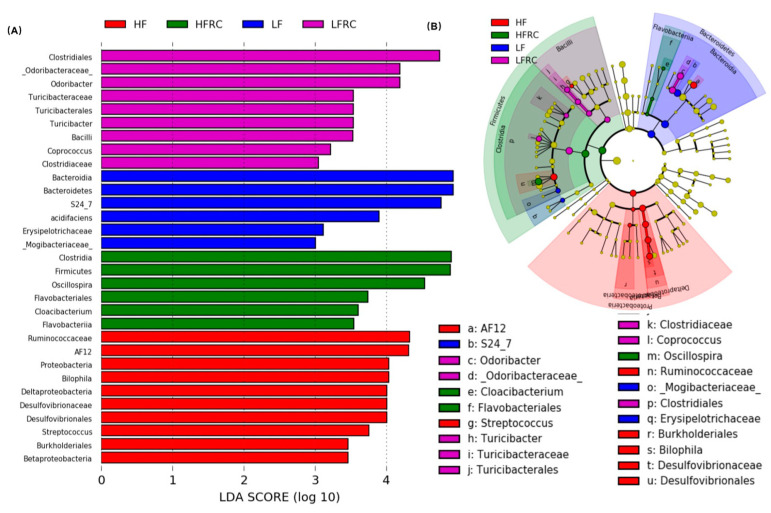
The comparison of alterations in cecal microbiota at the genus level was conducted using LEfSe analysis. (**A**) The LDA score histogram showed the effect size of each difference-rich feature between groups (only groups with significant LDA scores (log10) > 3); (**B**) Classification branch diagram with rich differences represented by the outermost ring gate and the innermost ring genus. Circle sizes correspond to their relative abundances.

**Figure 6:**
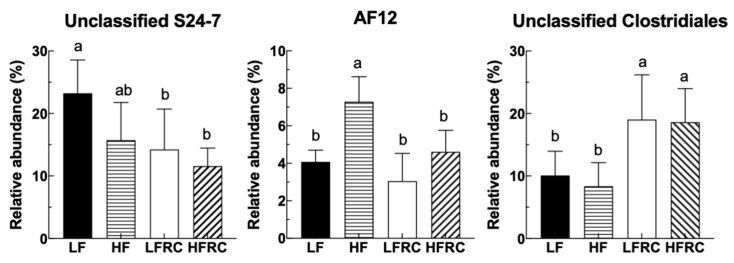
Intake of RC had significant effects on unclassified genera of *S27-4*, *AF12*, and unclassified genera of *Clostridium*. Data are expressed as mean ± SD, which are denoted by different letters to indicate a significant difference at *p* ≤ 0.05.

The abundance data of the microbiome taxa were additionally examined through linear discriminant analysis with effect size (LEfSe), a method that identifies distinct bacterial taxa based on their statistical significance and biological relevance [31]. As shown in Figure 5, in the four diet groups, a total of 31 distinct bacterial taxa were identified, of which eight genera were identified as potential biomarkers with significant statistical and biological importance, based on their LDA scores (log10) > 3 (Table 1). Specifically, as shown in Figure 6, the most discriminative genera among the diet groups were unclassified *S24-7* in the LF group; *AF12*, unclassified *Ruminococcaceae*, and *Bilophila* in the HF group; *Odoribacter*, unclassifed *Clostridiales*, and *Coprococcus* in the LFRC group; and *Oscillospira* in the HFRC group. The abundance of unclassified *S24-7* decreased in the LF supplemented with RC (LFRC) group, while *AF12* decreased in the HFRC group compared to the HF group. Adding RC to the diet resulted in a higher presence of unclassified *Clostridiales* in both LF- and HF-diet compositions.

### 3.5. Relationship between Gut Microbiota Communities and Biochemical Indices in Mice

**Figure 7:**
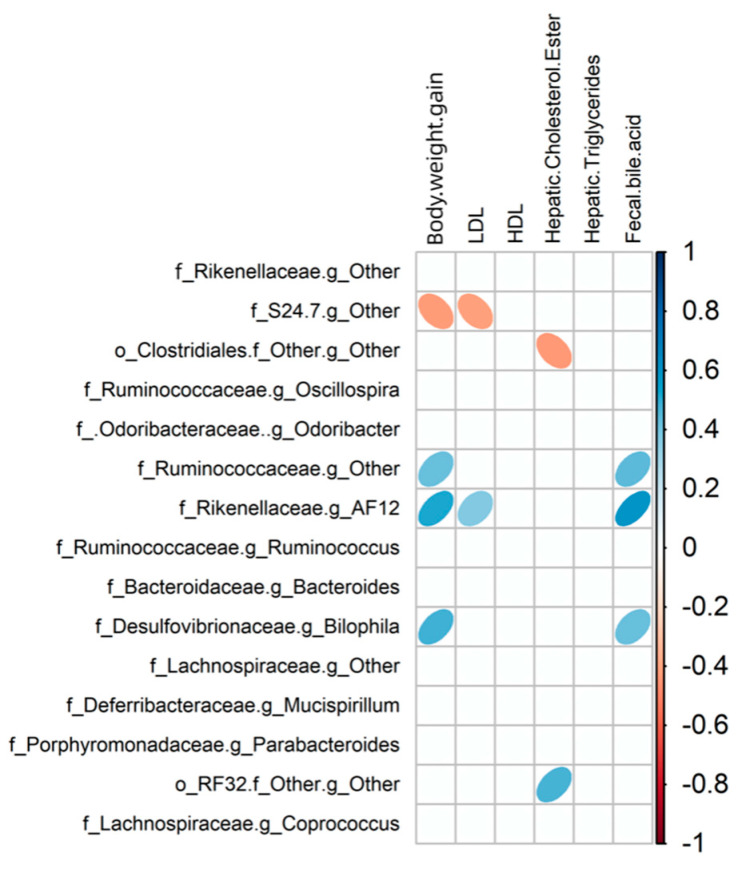
Pearson’s correlation matrix presents the interrelationships between dietary-influenced physiological parameters and the fifteen most prevalent genera, with adjustments for multiple testing via the Benjamini–Hochberg correction method. The genera are ranked by abundance. Blue ellipses denote positive correlations; whereas red ellipses indicate negative correlations.

The strength of the linear relationship between two variables can be measured using Pearson’s correlation analysis. In this work, this method was employed to evaluate the correlation between the gut microbiota communities and biochemical indices influenced by RC in mice. To represent the communities of gut microbiota, the selection was made for the top 15 genera, which made up around 98% of the bacterial sequence. Other factors that contributed to diet-induced obesity, including weight gain, plasma lipoprotein levels, hepatic cholesterol ester, and triglycerides, as well as bile acid in the feces, were used. As shown in Figure 7, to begin with, there was a negative correlation between *S24-7* and the index of body weight gain. In contrast, *Ruminococcaceae*, *AF12*, and *Bilophila* were positively correlated with body weight index and fecal bile acid. In addition, low-density lipoprotein levels (LDL) were negatively correlated with *S24-7* and positively correlated with *AF12*. Hepatic cholesterol ester was negatively correlated with unclassified *Clostridiales* and positively correlated with *RF32*. Also, none of the selected groups of bacteria correlated with either high-density lipoprotein levels (HDL) or hepatic triglycerides.

### 3.6. Effect of RC on the Gut Microbial Ecological Network

**Figure 8:**
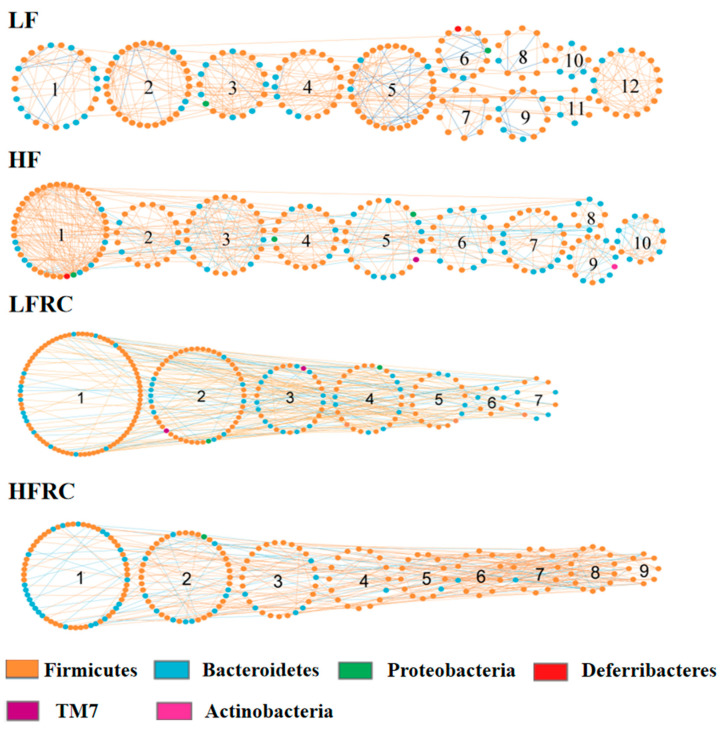
The depiction of the molecular ecological network of the gut microbiota is shown for different dietary regimes, including LF, HF, LFRC, and HFRC. Each node symbolizes an OTU, with the node’s color signifying different phyla. A positive interaction between two nodes is represented by a red line; whereas a negative interaction is indicated by a blue line. Modules are numbered in a certain order.

Furthermore, aside from alterations in species prevalence, the impact of various dietary interventions on the gut microbiota can be investigated using Molecular Ecological Network Analysis (MENA) to uncover co-occurrence patterns among species in microbial communities and their reaction to environmental changes. This is achieved by constructing networks based on the random matrix theory (RMT). As shown in Table S3, the LF group network consisted of 247 nodes (individual OUT/bacteria), 416 links (interactions), and 23 modules (community); the HF group network consisted of 245 nodes, 483 links, and 23 modules; the LFRC group network consisted of 233 nodes, 314 links, and 38 modules; and the HFRC group network consisted of 217 nodes, 406 links, and 27 modules. Hence, the addition of RC in the diet alters the number of interactions and communities in the ecological network. Modules with more than 5 nodes in each diet group were numbered, and the network diagrams are shown in Figure 8. The inclusion of RC changes the number of participating OUT, the interaction, and the communities. Modules 12, 10, 7, and 9 in LF, HF, LFRC, and HFRC groups, respectively, with a higher proportion of positive correlation among nodes supporting the taxa were more complementary or cooperative.

Bacteria (nodes) assume different roles in the network topology and vary in importance. Typically, determining the intra-module connectivity (Zi) and inter-module connectivity (Pi) of the network nodes is useful for identifying important nodes in the network. As shown in Figure S1, several nodes that act as connectors to connect different modules were identified. The LF group consists of six OTUs: OTU 380534, OTU 1108453, OTU 341713, OTU 387615, OTU 340853, and OTU 1517779. These OTUs belong to the genera *S24-7*, *Clostridiales*, *Ruminococcaceae*, *Oscillospira*, *Lachnospiraceae*, and *Rikenellaceae*. Additionally, OTU 390633 of the *S24-7* genus serves as the module center. Four OTUs in the HF group, namely OTU 2315700, OTU 404691, OTU 264657, and OTU 346804, belong to *Ruminococcus*, *S24-7*, and *Ruminococcaceae*, respectively, with OTU 340853 of *Lachnospiraceae* being used as module center. The LFRC group had one OTU, OTU 421792, which belonged to the genus *S24-7*, and OTU 263705 of *Peptococcaceae* was used as the module center. There were five OTUs in the HFRC group, OTU 268043, OTU 265940, OTU 268971, OTU 343906, and OTU 1109539. They belong to *Ruminococcus*, *Ruminococcaceae*, *Lachnospiraceae*, and *Lachnospiraceae*, respectively. OTU 278753 of *Clostridiales* was used as the module center. Also, no OTU appeared to serve as a network center (Figure S1).

The significant correlation between node connectivity and physiological traits based on OTU significance (GS) in the microbial coexistence network is shown in Table 2. The connectivity of nodes in the molecular ecological networks (MENs) of these four dietary groups correlated with weight gain, low-density lipoprotein (LDL), high-density lipoprotein (HDL), very-low-density lipoprotein (VLDL), hepatic cholesterol ester, hepatic free cholesterol, hepatic triglycerides, and fecal bile acid. There is a clear correlation between GS levels in physiological traits. Furthermore, within the MENs of the LF and HF groups, the OTUs originated from *Bacteroidaceae* and *Bacteroides*. These OTUs were strongly associated with cholesterol metabolism, including fecal bile acid and hepatic free cholesterol. Notably, *Bacteroides* exhibited the lowest *p*-value. The inclusion of RC could potentially enhance the population of advantageous microorganisms. The LFRC group consisted of OTUs originating from *Oscillospira*, *Ruminococcus*, and *Gnavus*, which had a strong correlation with hepatic free cholesterol and LDL. In contrast, the HFRC diet group was significantly different from the LFRC diet group, in which *Lachnospiraceae*, *S24-7*, *Coprococcus*, and *Oscillospira* were all closely associated with hepatic triglycerides.

The response of each module to a physiological trait was further evaluated and listed in Table 3. Three modules in the LF group displayed a positive correlation with the augmentation of body weight, hepatic levels of free cholesterol, and concentrations of bile acid in feces, whereas one module demonstrated a negative correlation with free cholesterol levels in the liver. Within the HF group, two modules showed a negative correlation with weight gain and hepatic free cholesterol, while four modules exhibited a negative correlation with VLDL, hepatic free cholesterol, and hepatic triglycerides, but a positive correlation with VLDL and hepatic triglycerides. The relationship between modules and physiological characteristics underwent significant changes in the LFRC and HFRC dietary groups. In both LFRC and HFRC groups, hepatic free cholesterol, HDL, and hepatic triglycerides showed a negative correlation with four modules.

## 4. Discussion

In the current research, various new findings were discovered regarding the consumption of red cabbage and its impact on the gut microbiome in a rodent model. Firstly, we found that dominant bacteria in the mouse feces responded rapidly to diet change 24 h after the introduction of experimental diets, similar to previous literature examining responses of the gut microbiome in humans [15]. These results support the utility of the mouse model to emulate human responses toward a short-term dietary intervention. Interestingly, short-term supplements of RC caused alterations in fecal bacteria in the LF matrix but not in the HF diet. The relative abundance of important genera, including *Bifidobacteria*, *Lactobacillus*, and *Akkermansia muciniphila*, was decreased in the feces of mice supplemented with RC in the LF-diet matrix; however, these alterations in fecal microbiota were not found in the cecal microbiota of mice fed red cabbage for 8 weeks. *Bifidobacteria* and *Lactobacillus* are common probiotic strains that are enriched by dietary sources such as fruits and vegetables [32,33,34]. The bacterium *A. muciniphila* has been known to reduce the risk of obesity by regulating metabolism and energy hemostasis. Moreover, *Enterobacteriaceae*, which contains many familiar pathogens, was also found to be reduced in the LFRC group. It is unclear how the above short-term changes of gut microbiota induced by RC consumption occurred. There are many phytochemicals, such as glucosinolates, in the RC. We have shown previously that glucosinolate-derived compounds can inhibit bacterial growth. The presence of those compounds may be sufficient to prevent specific bacterial growth. Alternatively, other bacteria may have been stimulated, therefore altering the bacterial profile in the feces. The lack of effect in the HF group may be explained by the presence of fat which may serve as the dominant factor in maintaining the bacterial profile, and the presence of phytochemicals may not be sufficient to alter that. Further studies are necessary to test these hypotheses.

The second interesting observation is the relatively muted effect of RC on the microbiome diversities in the cecal samples, i.e., longer-term exposure as compared to short-term analysis. The principal component analysis (PCoA) visualized significant differences in cecal microbial diversity between the LF and HF groups, while RC consumption resulted in an overlap between the LFRC and HFRC groups, suggesting that RC reduced the differences in the microbial community between the LF and HF groups. However, we found that RC caused only a slight, though not significant, increase in microbial species richness and evenness. Hence, the general profiles of the gut microbiome were not dramatically affected by supplementation with RC as seen in the short-term analysis. It would appear that the animal’s microbiome adapted from the initial short-term changes after 8 weeks on the RC diet.

Thirdly, at the phylum level, the effect of RC on the composition of cecal microbiota in mice was slightly different from that of fecal microbiota in mice following the 24 h meal. In addition to the increased F/B ratio, the RC consumption resulted in a significant suppression of the HF-diet-induced increase in Proteobacteria. It is well known that Proteobacteria is the phylum most affected by diets rich in fat, sugar, and animal protein, and is associated with the metabolic and inflammatory state of the body [35,36]. Moreover, specific bacterial families, such as *S24-7*, an unclassified family in *Clostridiates* and *Deferribacteraceae*, were affected by RC both in the LF diet and HF diet, further indicating the regulatory role of RC on the gut microbiota. However, it is worth noting that the effect of RC on microbial composition at the phylum level seems to be related to the dietary matrices. For instance, the increase in Firmicutes and the decrease in Bacteroidetes as a result of RC were significant in the HF matrix but not the LF matrix. Thus, the effect of food on gut microbial composition is complex and may be influenced by the interactions of other dietary elements ingested together.

Fourthly, specific genera that distinguished between different dietary groups were pinpointed as predictive biomarkers for 8-week treatment outcomes using LEfSe analysis. We found that the reductions of important genera such as *Bifidobacteria*, *Lactobacillus*, and *Akkermansia muciniphila* in fecal microbiota of the LFRC group were no longer significant in cecal microbiota of mice after 8 weeks of treatment. The differences in fecal and cecal microbiota in mice might be a consequence of mouse adaptation to food intake and environment, which warrants further elucidation. Furthermore, the link between these microbial markers and obesity-associated risk factors was reinforced by Pearson’s correlation analysis. As an example, Genus *AF12*, which was recognized as a biomarker in the HF-diet group, exhibited a favorable association with the increase in body weight, levels of LDL, and fecal bile acid in mice. The increased relative abundance of *AF12* prompted by an HF diet was notably reduced by the addition of RC. Additionally, an unclassified genus within the *Clostridiales* order, which was augmented by RC supplementation, displayed a negative correlation with hepatic cholesterol ester concentrations in mice. These observations suggest that RC may counteract the rise in markers linked to gut inflammation and obesity-related conditions, including body weight, LDL, fecal bile acid, and hepatic free cholesterol, via alterations in gut microbiota composition. RC reduced the presence of an unclassified genus in the *S24-7* family, which was found to be a biomarker in the LF diet and showed an inverse relationship with weight gain and LDL levels, in both LF and HF diets. This indicates that this bacterium could serve as an indicator of RC consumption and may contribute to the health-promoting effects of RC.

Ultimately, the analysis of the correlations within microbial co-occurrence networks provided greater clarity on how the topology of microbial networks is related to physiological features. Selected bacterial taxa in the microbial network of each dietary group were found to be closely associated with specific physiological characteristics. *Bacteroidaceae* and *Bacteroides* identified in the LF- and HF-diet groups were found to be associated with cholesterol metabolism. These bacteria are likely involved in the uptake of dietary fat, which in turn regulates the metabolism of fecal bile acid and hepatic free cholesterol. Specifically, the OTUs from *Bacteroides* were linked with fecal bile acid and hepatic free cholesterol levels in both the LF- and HF-diet groups, suggesting their role in dietary fat processing and cholesterol metabolism. The addition of RC seemed to diversify the bacterial involvement in cholesterol metabolism, as bacteria other than *Bacteroides* were also correlated with these metabolic indicators in the LFRC and HFRC groups. The correlation analysis between eigengenes based on modules and physiological characteristics in microbial co-occurrence networks provided additional support for this concept. The phylogenetic MEN for the four experimental groups all exhibited strong correlations between their submodules and physiological parameters, with notable differences in submodule responses to cholesterol metabolism when comparing diets with and without RC. Overall, our results show that RC could alter microbial interaction networks but the exact mechanisms of how these changes can modulate biological endpoints need further exploration.

We recently reported an effect of red cabbage microgreen, a young version of red cabbage, on the gut microbiome [36]. Although RC microgreens and mature RC represent different growing stages of RC [23], their effect on the microbiome appears to be different. RC microgreen seemed to elicit more robust changes in alpha diversity as more indices were found to be changed when animals consumed RC microgreen rather than the mature RC [36]. Additionally, mature RC consumption led to changes in *Deferribacteraceae* but not for the consumption of RC microgreens. Given the composition of RC microgreens and mature RC are different [23], these differences in the gut microbiome may be reflective of composition differences in the RC growing stages. However, there are also similarities in the gut microbiome in the animals, elicited by consuming RC microgreens and mature RC. The abundance of the bacteria family *S24-7* was reduced when animals consumed RC microgreens [36] or mature RC. In contrast, the unclassified *Clostridiales* abundance was increased when animals consumed RC microgreen [36] or mature RC. These data suggest common composition; for example, fiber may determine the phenotype displayed. However, further experiments are needed to elucidate the precise component and substantiate the utilities of these bacteria changes as biomarkers of intake.

## 5. Conclusions

In conclusion, our hypothesis was confirmed that RC can modulate the gut microbiome of mice fed in both LF and HF diets, and the effect appeared to involve specific microbial compositions as well as microbial interaction. This work additionally underscores that certain bacteria could act as dietary biomarkers, exemplified by unclassified *S24-7* for the LF diet, *AF12* for the HF diet, and an unclassified member of *Clostridiales* for diets inclusive of RC. These bacteria were associated with obesity-related risk factors and were affected by RC supplementation. However, further studies with more comprehensive microbiome and metabolome data are needed to elucidate the causal biological pathways by which RC alleviates diet-induced obesity through the modulation of gut microbiota. Additionally, components of the dietary matrix, such as fat, may influence the effect of dietary RC on gut microbiota and warrant further validation.

## Figures and Tables

**Table 1:** Percentage relative abundance of key biomarkers as determined with LEfSe analysis.

Phylum/Class; Order; Family	Genus	Diet			
		LF	HF	LFRC	HFRC
**Bacteroidetes**					
Bacteroidia; Bacteroidales; S24-7	Other	23.24 ± 5.32 ^a^	15.74 ± 6.01 ^ab^	14.23 ± 6.49 ^b^	11.59 ± 2.87 ^b^
Bacteroidia; Bacteroidales; Rikenellaceae	AF12	4.08 ± 0.62 ^b^	7.29 ± 1.33 ^a^	3.05 ± 1.48 ^b^	4.61 ± 1.15 ^b^
Bacteroidia; Bacteroidales; [Odoribacteraceae]	Odoribacter	7.60 ± 2.26 ^ab^	6.52 ± 1.88 ^b^	9.73 ± 2.49 ^a^	6.22 ± 1.60 ^b^
**Firmicutes**					
Clostridia; Clostridiales; Ruminococcaceae	Oscillospira	10.17 ± 1.97 ^b^	14.01 ± 2.74 ^ab^	9.45 ± 2.99 ^b^	16.80 ± 4.34 ^a^
Clostridia; Clostridiales; Ruminococcaceae	Other	5.17 ± 1.52 ^b^	8.70 ± 1.80 ^a^	4.19 ± 1.57 ^b^	7.61 ± 1.66 ^a^
Clostridia; Clostridiales; Other	Other	10.10 ± 3.87 ^b^	8.36 ± 3.78 ^b^	19.04 ± 7.16 ^a^	18.62 ± 5.36 ^a^
Clostridia; Clostridiales; Lachnospiraceae	Coprococcus	0.24 ± 0.05 ^a^	0.34 ± 0.26 ^a^	0.55 ± 0.22 ^a^	0.53 ± 0.22 ^b^
**Proteobacteria**					
Deltaproteobacteria; Desulfovibrionales; Desulfovibrionaceae	Bilophila	2.52 ± 0.60 ^a^	4.03 ± 0.47 ^a^	1.81 ± 0.66 ^b^	3.39 ± 0.64 ^b^

^a^ gray highlight shows the biomarkers assigned to the corresponding group. ^b^ Tukey’s multiple comparisons test was used to determine the differences between the two groups. The data are displayed as mean ± SD, with distinct letters indicating significant variations, signifying a significant distinction at *p* ≤ 0.05.

**Table 2:** The correlation between physiological traits and OTU significance in microbial symbiosis network.

Diet Group	Physiological Traits	Bacterial Taxa (Rank)	r ^a^	*p*-Value ^b^
LF	Fecal bile acid	Bacteroidaceae (Family)	0.741	0.004
	Fecal bile acid	Bacteroides (Genus)	0.741	0.002
HF	Hepatic free cholesterol	Bacteroidaceae (Family)	0.684	0.003
	Hepatic free cholesterol	Bacteroides (Genus)	0.684	0.001
LFRC	Hepatic. free. cholesterol	Oscillospira (Genus)	0.316	0.046
	LDL	[Ruminococcus] (Genus)	0.790	0.017
	LDL	Gnavus (Species)	0.790	0.017
HFRC	Hepatic triglycerides	Lachnospiraceae (Family)	0.269	0.003
	Hepatic triglycerides	S24-7 (Family)	0.243	0.036
	Hepatic triglycerides	Coprococcus (Genus)	0.718	0.017
	Hepatic triglycerides	Oscillospira (Genus)	0.134	0.048

^a^ Correlation coefficient based on the Mantel test. ^b^ Significance (probability) of the Mantel test.

**Table 3:** Associations between node interconnectivity within modules and physiological traits in microbial co-occurrence networks. (^a^ Correlation coefficient based on the Mantel test. ^b^ The significance (probability) of the Mantel test).

Diet Group	Module	Physiological Traits	r ^a^	*p*-Value ^b^
LF	9	Body weight	0.84	0.02
	7	Hepatic cholesterol ester	0.88	0.008
	7	Hepatic free cholesterol	−0.92	0.003
	1	Fecal bile acid	0.81	0.03
HF	10	Body weight	−0.81	0.03
	2	VLDL	0.83	0.02
	9	VLDL	0.89	0.007
	8	Hepatic cholesterol ester	−0.79	0.04
	6	Hepatic free cholesterol	0.84	0.02
	7	Fecal bile acid	0.79	0.03
LFRC	7	Hepatic free cholesterol	−0.72	0.05
HFRC	6	HDL	−0.74	0.04
	8	Hepatic triglycerides	−0.80	0.02
	5	Hepatic free cholesterol	−0.73	0.04

## Data Availability

Data is contained within the article.

## Supplementary Materials

The following supporting information can be downloaded at: https://www.mdpi.com/article/10.3390/foods13010085/s1, Figure S1: The Zi-Pi diagram shows the distribution of the topological role of OTU in the network; Table S1: Primer sequence used for microbial analysis by real-time PCR; Table S2: Relative abundance (%) of the bacterial taxa at phylum level in cecal samples of mice grouped by diet; Table S3: The topological properties of the global network are inferred by using the network pipeline based on random matrix theory (RMT) under various experimental conditions.
