# Supplementary material for: Red Cabbage Modulates Composition and Co-Occurrence Networks of Gut Microbiota in a Rodent Diet-Induced Obesity Model
Source: Foods. 2023 Dec 26;13(1):85. doi: 10.3390/foods13010085 (PMC10778922; doi:10.3390/foods13010085)
Supplement: Supplementary file 1 [file foods-13-00085-s001.zip › foods-2727611-supplementary.pdf]

Electronic Supplementary Information

Red Cabbage Modulates Composition and Co-Occurrence Networks of Gut Microbiota in a Rodent Diet-Induced Obesity Model

Table S1. Primer sequence used for microbial analysis by real-time PCR.

**Bacteria**	**Direction**	**Sequence (5′–3′)**
Bacteroidetes	Forward	GGARCATGTGGTTTAATTCGATGAT
	Reverse	AGCTGACGACAACCATGCAG
Firmicutes	Forward	GGAGYATGTGGTTTAATTCGAAGCA
	Reverse	AGCTGACGACAACCATGCAC
Prevotella	Forward	TCCTACGGGAGGCAGCAGT
	Reverse	CAATCGGAGTTCTTCGTG
Enterobacteriaceae	Forward	CATTGACGTTACCCGCAGAAGAAGC
	Reverse	CTCTACGAGACTCAAGCTTGC
Ruminococcus	Forward	GGCGGCCTACTGGGCTTT
	Reverse	CCA GGT GGA TAA CTT ATT GTG TTAA
Bifidobacteria	Forward	TCGCGTCYGGTGTGAAAG
	Reverse	CCACATCCAGCRTCCAC
Lactobacillus	Forward	GAGGCAGCAGTAGGGAATCTTC
	Reverse	GGCCAGTTACTACCTCTATCCTTCTTC
Akkermansia muciniphila	Forward	CAGCACGTGAAGGTGGGGAC
	Reverse	CCT TGCGGTTGGCTTCAGAT

Table S2. Relative abundance (%) of the bacterial taxa at phylum level in cecal samples of mice grouped by diet (LF: LF diet, HF: HF diet, LFRC: LF diet supplemented with RC powder, HFRC: HF diet sup-plemented with RC powder).

Phylum	Diet			
	LF	HF	LFRC	HFRC
Bacteroidetes	60. 00±2. 45	53. 47±6. 21	47. 31±10. 30	42. 6±6. 53
Firmicutes	34. 41±2. 19	39. 19±5. 92	42. 17±11. 50	50. 58±7. 34
Proteobacteria	3. 18±0. 61	4. 78±0. 26	2. 57±0. 61	3. 81±0. 68
Deferribacteres	2. 17±0. 78	2. 38±1. 24	4. 06±1. 43	2. 93±1. 09
TM7	0. 08±0. 04	0. 09±0. 11	0. 09±0. 09	0. 05±0. 04
Actinobacteria	0. 01±0. 00	0. 01±0. 00	0. 02±0. 01	0. 02±0. 01

Table S3. The topological properties of the global network are inferred by using the network pipeline based on random matrix theory (RMT) under various experimental conditions.

Network Indexes	LF	HF	LFRC	HFRC
Total nodes	247	245	233	217
Total links	416	483	314	406
Total modules	23	23	38	27
Modularity (M)	0. 819	0. 72	0. 772	0. 704
R square of power-law	0. 71	0. 80	0. 818	0. 825
Average degree (avgK)	3. 368	3. 94	2. 695	3. 742
Average clustering coefficient (avgCC)	0. 304	0. 32	0. 145	0. 17
Average path distance (GD)	8. 217	6. 97	6. 603	4. 905
Geodesic efficiency (E)	0. 166	0. 20	0. 2	0. 261
Harmonic geodesic distance (HD)	6. 039	4. 92	4. 992	3. 826
Maximal degree	13	16	12	15
Nodes with max degree	OTU 262625	OTU 1136443<br>OTU 1684221<br>OTU 329790<br>OTU 317633	OTU 263705	OTU 336691
Centralization of degree (CD)	0. 039	0. 05	0. 04	0. 053
Maximal betweenness	6815. 31	4996. 26	3378. 541	2751. 546
Nodes with max betweenness	OTU 262677	OTU 275366	OTU 275366	OTU 339031
Centralization of betweenness (CB)	0. 204	0. 15	0. 114	0. 11
Maximal stress centrality	48167	33304	20539	31008
Nodes with max stress centrality	262677	418501	275366	339031
Centralization of stress centrality (CS)	1. 451	1. 04	0. 713	1. 262
Maximal eigenvector centrality	0. 355	0. 31	0. 435	0. 31
Nodes with max eigenvector centrality	OTU 353012	OTU 317633	OTU 263705	OTU 277208
Centralization of eigenvector centrality (CE)	0. 335	0. 28	0. 416	0. 279
Density (D)	0. 014	0. 02	0. 012	0. 017
Reciprocity	1. 00	1. 00	1. 00	1. 00
Transitivity (Trans)	0. 359	0. 35	0. 182	0. 214
Connectedness (Con)	0. 78	0. 63	0. 515	0. 481
Efficiency	0. 987	0. 98	0. 985	0. 972
Hierarchy	0. 00	0. 00	0. 00	0. 00
Lubness	1. 00	1. 00	1. 00	1. 00

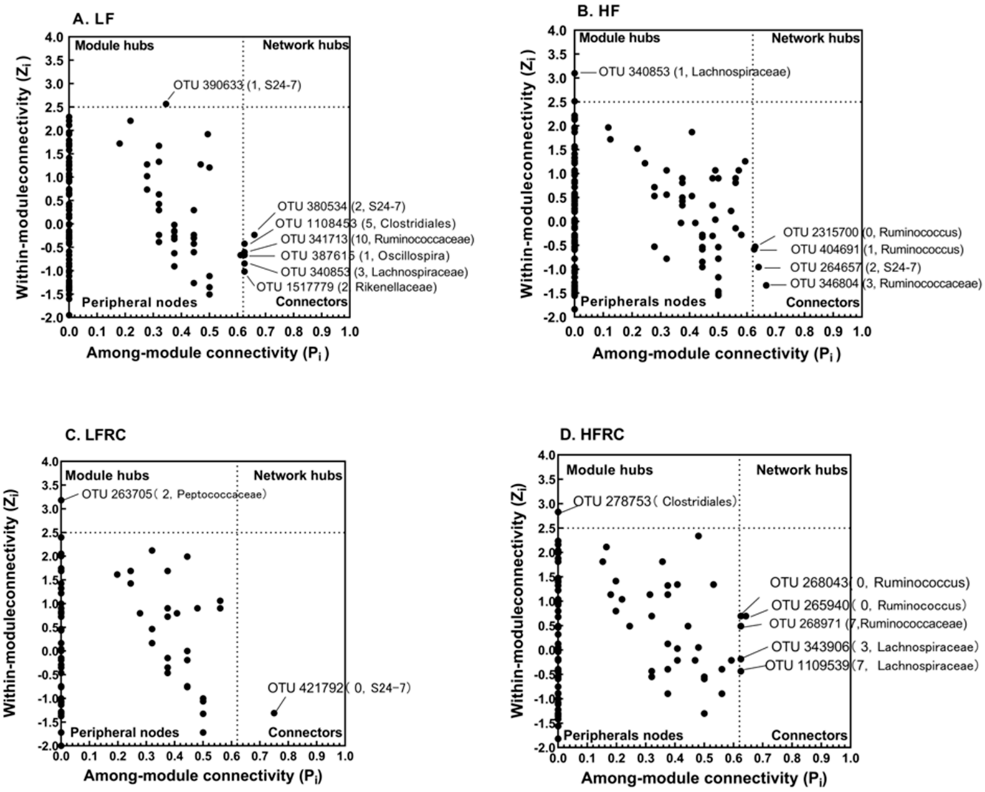

Figure S1. The Zi-Pi diagram shows the distribution of the topological role of OTU in the network. Each point represents an OTU under different dietary groups (LF: LF diet, HF: HF diet, LFRC: LF diet with RC powder, HFRC: HF diet with RC powder). According to the scatter diagram of in-tra-module connectivity (Zi) and inter-module connectivity (Pi), the topological function of each OTU is determined. The module hub and connector are marked with OTU number. In parentheses are the module number and phylogenetic associations.
